# Prevalence and risk factors of prenatal depression among pregnant women attending antenatal clinic at Adventist Hospital, Bekwai Municipality, Ghana

**DOI:** 10.1097/MD.0000000000028862

**Published:** 2022-03-11

**Authors:** Kwabena Acheanpong, Xiongfeng Pan, Atipasa Chiwanda Kaminga, Aizhong Liu

**Affiliations:** a Department of Epidemiology and Health Statistics, Xiangya School of Public Health, Central South University, Changsha Hunan, China; b Department of Public Health, Adventist University of Africa, Kenya; c Hunan Provincial Key Laboratory of Clinical Epidemiology, Xiangya School of Public Health, Central South University, Changsha, China.

**Keywords:** depression, Ghana, prenatal depression, prevalence, risk factors

## Abstract

Depression, arising in the perinatal period are a major health issue in low- and middle-income countries. However, little attention has been paid in the research of depression symptoms. This study aimed to estimate the prevalence and risk factors of depression during pregnancy.

A cross-sectional study was conducted in pregnant women attending antenatal clinic at Adventist Hospital in the Bekwai Municipality, Ghana, between February and May 2020. Information on sociodemographic, medical, and obstetric factors were collected from the antenatal booklet and prenatal depression symptoms was defined as a patient health questionnaire scores ≥10. Descriptive statistics, Chi-Squared test, and Fisher exact test were used to analyze dichotomous variables. Multivariate logistic regression model was applied to estimate the adjusted odds ratios (AOR) and 95% confidence interval (95% confidence interval [CI]) for risk factors associated with prenatal depression. All statistical analyses were performed using SPSS version 20.0.

The prevalence of prenatal depression in this study was 26.9% (95% CI; 24.6%–29.2%). Advance maternal age ≥35years (AOR = 1.49, 95% CI 1.05–2.11, *P* < .026) and low educational attainment (AOR 2.15, 95% CI 1.23–2.34, *P* < .007) were significantly higher among women with parental depression compared with maternal age <35years and higher educational attainment respectively. Similarly, nulliparous women (AOR = 4.93, 95% CI 1.60–15.16, *P* < .005), primiparous women (AOR = 5.42, 95% CI 1.76–16.71, *P* < .003) and multiparous women (AOR = 4.79, 95% CI 1.61–14.22, *P* < .005) were significantly higher among women with parental depression compared with grand multiparous woman (≥7 deliveries). Finally, prenatal depression was found to be significantly associated with hypertension in pregnancy (AOR = 1.71, 95% CI: 1.12–2.60, *P* < .013).

Depression during pregnancy is high in the study area and is significantly associated with advance maternal age, low educational attainment, parity less than 7 deliveries, and hypertension during pregnancy.

## 1 Introduction

Depression, anxiety, and somatic symptoms, known as common psychiatric illness, arising in the perinatal period, which commences at 22 completed weeks (154 days) of gestation and ends 7 completed days after birth are a major health issue in low- and middle-income countries.^[1]^ Depression is a typical disease that is widely spread across the population and is usually associated with extreme symptoms and loss of roles. Although the recent increase in care is promising, the lack of appropriate services is a major problem. The emphasis on screening and treatment expansion must be balanced by a corresponding focus on improving care efficiency. Depression symptoms are most common in women during pregnancy,^[2]^ possibly due to a combination of hormonal changes and a number of psychosocial factors.^[3]^ Given the primary role of care often played by women, antenatal depression can potentially have important implications for fetal growth and child development.^[4]^ Evidence suggests that females born to depressed mothers could have a higher risk of perinatal depression (a mood disorder that affect women during pregnancy and after childbirth) relative to those born to non-depressed mothers.^[5,6]^ At the individual level, in conjunction with antenatal depression (a form of clinical depression that affect women during pregnancy), the risk of low birth weight, preterm birth, intrauterine growth restriction, low Apgar score and pregnancy complications is considered to be higher.^[7–9]^ In addition, antenatal depression has been associated with neurodevelopmental, social and attachment difficulties and educational outcomes, malnutrition, respiratory disorders and a higher risk of developing later-life mental health disorders for infants.^[10–13]^ Depression during pregnancy can also affect maternal health, adherence to medical and psychological treatments, and increased risk behaviors such as substance use and misuse.^[14,15]^ However, there is a limited number of studies examining the associated factors of antenatal depression in Sub-Sahara Africa. Various studies have been conducted in Ghana on the prevalence of depression among women, ranging from 3.8% to 34.4%.^[16–19]^ But few of these studies have focused on prenatal depression. Despite the link between pregnancy depression and adverse birth outcomes, no data are available in the study area. The aim of this study was therefore to estimate prevalence and risk factors associated with depression during pregnancy at Adventist Hospital, Bekwai Municipality, Ghana.

## 2 Methods

### 2.1 Study design, setting, and participants

A cross-sectional study was carried out to estimate the prevalence of depression and factors associated with women attending Antenatal Clinic at Adventist Hospital, Bekwai Municipality, Ghana. The Municipality is located in the southern part of the Ashanti region, lies within 6° 00’N - 6°30 ‘N and Longitudes 1°00 W and 1° 35 W and it covers a total land area of about 633 sqkm. In other parts of the Municipality, however, human activities, particularly farming and timber extraction, have reduced the primary forest to secondary forests. The population of the Municipality according to the 2010 Population and Housing Census stands at 118,024 with 55,615 males 62,409 females and nearly 97,277 (82.4%) reside in rural areas. The eligibility criteria included women above or equal to 18 years of age, pregnant at gestational age 18 to 33 weeks, permanent residents in the target area and having no psychological treatment or obstetric complication in the current pregnancy. Data collection began in February and ended in May 2020.

### 2.2 Ethical considerations

Ethical approval was obtained from Research and Ethics Review Committee of the Department of Epidemiology and Health Statistics, Central South University, China prior to the commencement of the study. A written informed consent was obtained from each study participant after the intent and significance of the study was explained.

### 2.3 Sample size and power analysis

The sample size for this study was calculated using a single population proportion formula with a 95% confidence interval (CI), a 5% margin error and an assumption that 50% of pregnant women were depressed. By adding 10% for non-response, the minimum sample size required for this study was calculated as n = 400 participants. Systematic random sampling method was used to recruit a total of 1433 study participants from their Antenatal Care visit sequence during the study period.

### 2.4 Outcome measure

Prenatal depression was the outcome of study, which was measured using the patient health questionnaire-9 (PHQ-9). PHQ-9 is one of the most validated tools in mental health and can be a powerful tool to assist clinicians with diagnosing depression and monitoring treatment response. The 9 items of PHQ-9 are based directly on the 9 diagnostic criteria for major depressive disorder in the Diagnostic and Statistical Manual of Mental Disorders, Fourth Edition. Each of the 9 Diagnostic and Statistical Manual of Mental Disorders, Fourth Edition criteria was scored as “0” (not at all) to “3” (nearly every day), with a range of 0 to 27. Prenatal depression in this study was defined as PHQ-9 score ≥10. PHQ-9 score of 10 or higher can detect more cases of major depression than the PHQ determination of major depression originally described by Spitzer et al in 1999.^[20]^ PHQ-9 has a good internal consistency (Cronbach's alphas range from 0.83 to 0.89),^[21]^ good inter-rater reliability ranges between 0.83 and 0.92^[22]^ and adequate test-retest reliability correlation of 0.84 between administrations done within 48 hours.^[21]^ A PHQ-9 score cutoff of 10 yielded sensitivity and specificity rates of 85% and 84%, respectively, for a depression diagnosis and 75% and 88% for a sub-diagnosis, respectively.^[23]^

### 2.5 Independent variables

Major socio-demographic, obstetric, and clinical factors were collected from antenatal book at enrolment. Socio-demographic included in this study were maternal age (18–25, 26–34, and ≥35 years), maternal education (tertiary, senior high school, Junior high School, and primary school and below), employment status (salary worker, trader, apprenticeship, farmer, hairdresser, and unemployed), and pre-pregnancy Body Mass Index (or BMI, weight in kg/height in m^2^, which was categorized as underweight (<18.5), normal weight (≤18.5–24.9), overweight (≥ 25–29.9), and obese (BMI ≥ 30).

Obstetric and Clinical factors included in this study were gravida (primigravida was defined as been pregnant for the first time. for the purpose of this study “multigravida” was defined as been pregnant 2 to 4 times or “grand multigravida” was defined as been pregnant 5 times or more), parity (“nulliparity” was defined as zero delivery, “primiparity” was defined as 1 delivery, for the purpose this study “multiparity” was defined as 2 to 6 deliveries or “grand multiparity” was defined as ≥7 deliveries), gestational weeks at recruitment (<28weeks and ≥28weeks), syphilis (yes/no), hepatitis B (yes/no), human immunodeficiency virus (yes/no), malaria (yes/no), sickling cell trait (yes/no), hypertension in pregnancy (yes/no, which was defined as systolic blood pressure ≥140 mm Hg and/or diastolic blood pressure ≥90 mm Hg), and anemia status (yes/no, which was defined as a hemoglobin concentration of less than 110 g/L (less than 11 g/dL) in venous blood).

### 2.6 Statistical analysis

All statistical analyses were performed using SPSS version 20.0 (IBM Inc). Continuously distributed variables were presented as means and standard deviation (SD) categorical variables were presented as proportions (%). Participants were divided into 2 groups based on PHQ-9 score: a non-depressed group (PHQ score <10), and a depressed group (PHQ score ≥10). The independent variables were categorized to analyze the association between each independent and outcome variable using a bivariate analysis to calculate the Crude Odd Ratio (COR) with 95% CI. Those variables that were associated at a *P* value of <.7 in the bivariate analysis were entered into a multivariate logistic regression model to calculate the Adjusted Odd Ratio (AOR) and to eliminate the effects of confounding. Variables with a *P* value of <.05 in the multivariate analysis were considered to be significant.

## 3 Results

### 3.1 Characteristics of the study participants

A total of 1433 pregnant women were included in the study with the mean age of 27.98 ± 6.55years (range 18–47). Age records were divided into 3 groups: 18–25 years (36.0%), 26–34 years (45.7%), and ≥35 years (18.4%). One hundred eighty one (50.3%) had completed junior and 30% had low level of education (no formal or primary school education). With regard to obstetric characteristics, 992(69.2%) were in their second trimester, primigravida accounted for 314(21.9%) while both multigravidas and grand multigravidas accounted for 1119(78.6%) of pregnant women. Nulliparas, primiparas, multiparas accounted for 411(28.7%), 291(20.3%), and 730(51.0%) of pregnant women as summarized in Table 1.

**Table 1:** Prevalence of prenatal depression among pregnant women by sociodemographic, obstetric, and clinical characteristics of pregnant women (N = 1433).

		Depression		
Variable	N (%) Screen	No	Yes	*P* value
Age in years				**.035**
18–25	513 (36.0)	374 (35.9)	139 (36.2)	
25–34	651 (45.7)	492 (47.2)	159 (41.4)	
≥35	262 (18.4)	86 (22.4)	176 (16.9)	
Education				**.001**
Tertiary	88 (6.1)	68 (6.5)	20 (5.2)	
Senior High School	197 (13.7)	165 (15.8)	32 (8.3)	
Junior High School	636 (44.4)	483 (46.1)	153 (39.6)	
Primary & below	512 (35.7)	331 (31.6)	181 (46.9)	
Employment status				.132
Salary worker	60 (4.2)	41 (3.9)	19 (4.9)	
Trader	497 (34.7)	354 (33.8)	143 (37.0)	
Apprenticeship	133 (9.3)	100 (9.6)	33 (8.5)	
Farmer	74 (5.2)	50 (4.8)	24 (6.2)	
Hairdresser	286 (20.0)	203 (19.4)	83 (21.5)	
Unemployed	383 (26.7)	299 (28.6)	84 (21.8)	
Weeks of gestation at recruitment				.537
<28weeks	992 (69.2)	720 (68.8)	272 (70.5)	
≥28weeks	441 (30.8)	327 (31.2)	114 (29.5)	
Gravida				0.518
Primigravida	314 (21.9)	224 (21.4)	90 (23.3)	
Multigravida	726 (50.7)	528 (50.4)	198 (51.3)	
Grand multigravida	393 (27.4)	295 (28.2)	98 (25.4)	
Parity				**.037**
Nulliparity	411 (28.7)	298 (28.5)	113 (29.3)	
Primiparity	291 (20.3)	209 (20.0)	82 (21.2)	
Multiparity	683 (47.7)	496 (47.4)	187 (48.4)	
Grand multiparity	47 (3.3)	43 (4.1)	4 (1.0)	
Blood type				.930
Type A	177 (12.4)	131 (12.5)	46 (11.9)	
Type B	213 (14.9)	152 (14.5)	61 (15.8)	
Type AB	85 (5.9)	63 (6.0)	22 (5.7)	
Type O	958 (66.9)	701 (67.0)	257 (66.6)	
Syphilis				.390
No	1380 (96.3)	1011 (96.6)	369 (95.6)	
Yes	53 (3.7)	36 (3.4)	17 (4.4)	
Hepatitis B surface antigen (HBsAg) status				.211
No	1356 (94.6)	986 (94.2)	370 (95.9)	
Yes	77 (5.4)	61 (5.8)	16 (4.1)	
PMTCT of HIV				.235
No	1424 (99.4)	1042 (99.5)	382 (99.0)	
Yes	9 (0.6)	5 (0.5)	4 (1.0)	
Sickling cell status				.615
Negative	1301 (90.8)	953 (91.0)	348 (90.2)	
Positive	132 (9.2)	94 (9.0)	38 (9.8)	
Malaria infection at registration				.325
No	1267 (88.4)	931 (88.9)	336 (87.0)	
yes	166 (11.6)	116 (11.1)	50 (13.0)	
Hypertension at registration				**.016**
No	1321 (92.2)	976 (93.2)	345 (89.4)	
Yes	112 (7.8)	71 (6.8)	41 (10.6)	
Anemia at registration				.596
Severe Anemia (<7.0 g/dL)	10 (0.7)	9 (0.9)	1 (0.3)	
Moderate Anemia (7.0–9.9g/dL)	348 (24.3)	257 (24.5)	91 (23.6)	
Mild Anemia (10.0–10.9 g/dL)	521 (36.4)	382 (36.5)	139 (36.0)	
No Anemia (≥11 g/dL)	554 (38.7)	399 (38.1)	155 (40.2)	
Body mass index (BMI) group				.162
Underweight	24 (1.7)	16 (1.5)	8 (2.1)	
Normal weight	670 (46.8)	483 (46.2)	187 (48.4)	
Overweight	443 (30.9)	340 (32.5)	103 (26.7)	
Obese	295 (20.6)	207 (19.8)	88 (22.8)	

Definition: For the purpose of this study “Grand Multigravida” was defined as was been pregnant 5 times or more; “Grand Multiparity” was defined as 7 times or more deliveries. PMTCT = prevention of mother-to-child transmission, HIV = human immunodeficiency.

### 3.2 Prevalence of prenatal depression

Patient health questionnaire-9 scale assessment were done to all the study subjects to check for depression. The mean PHQ-9 scores were (Mean ± SD) 8.64 ± 2.43. The results indicated 386 women 26.9% (95% CI; 24.6%–29.2%) with PHQ-9 scores of 10 or higher, with 348 cases (24.5%) considered moderate risk, and 37 (2.6%) high risk.

### 3.3 Association of prenatal depression with sociodemographic, obstetrics, and clinical factors

All variables were analyzed using binary logistic regression to evaluate factors associated with prenatal depression among the study participants as summarized in Table 2. Association with advance maternal age ≥35years was not significant on bivariate analysis but not in multivariate logistic regression (COR = 1.32; 95% CI: 0.95–1.82, *P* > .097). Primary school or no formal education was found to be a risk factor of prenatal depression among the women (COR = 1.98; 95% CI: 1.10–3.16, *P* < .022). Nulliparous women [COR = 4.08 (95% CI; 1.43–11.62, *P* < .009)]; primiparous women (COR = 4.22: 95% CI 1.47–12.12, *P* < .008); and multiparous women were about 4 times (COR = 4.05: 95% CI 1.44–11.45, *P* < .008) more likely to have prenatal depression compared to women with grand multiparous., Finally, participants who had hypertension during pregnancy were 1.68 times (COR = 1.63(95% CI; 1.09–2.45, *P* < .017) more likely to have prenatal depression compared to participants without hypertension in pregnancy. There was also no evidence of association between prenatal depression and employment status, gravida, syphilis, hepatitis B surface antigen (HBsAg), prevention of mother-to-child transmission of human immunodeficiency virus, sickling cell status, malaria infection status, weeks of gestation age at recruitment, anemia in pregnancy and BMI groups.

**Table 2:** Binary logistic regression analysis of factors associated with prenatal depression (N = 1433).

Variables	Frequency	COR (95% CI)	*P* value
Age in years			
<25	513 (36.0)	1	–
25–34	651 (45.7)	0.87 (0.68–1.13)	.300
≥35	262 (18.4)	1.32 (0.95–1.82)	.097
Education			
Tertiary	88 (6.1)	1	–
Senior High School	197 (13.7)	0.66 (0.35–1.23)	.192
Junior High School	636 (44.4)	1.08 (0.63–1.83)	.784
Primary & below	512 (35.7)	1.98 (1.10–3.16)	.022
Employment status			
Salary worker	60 (4.2)	1	–
Trader	497 (34.7)	0.87 (0.49–1.55)	.641
Apprenticeship	133 (9.3)	0.61 (0.33–1.10)	.099
Farmer	74 (5.2)	0.71 (0.36–1.39)	.322
Hairdresser	286 (20.0)	1.04 (0.50–2.15)	.925
Unemployed	383 (26.7)	0.88 (0.48–1.61)	.683
Weeks of Gestational age at recruitment			
<28weeks	992 (69.2)	1	
≥28weeks	441 (30.8)	0.92 (0.72–1.19)	.537
Gravida			.518
Primigravida	314 (21.9)	1	
Multigravida	726 (50.7)	0.93 (0.70–1.25)	.646
Grand multigravida	393 (27.4)	0.83 (0.59–1.16)	.266
Parity			**.065**
Nulliparity	411 (28.7)	4.08 (1.43–11.62)	.009
Primiparity	291 (20.3)	4.22 (1.47–12.12)	.008
Multiparity	683 (47.7)	4.05 (1.44–11.45)	.008
Grand multiparity	47 (3.3)	1	–
Syphilis			
No	1380 (96.3)	1	–
Yes	53 (3.7)	1.29 (0.72–2.33)	.391
Hepatitis B surface antigen (HBsAg) status			
No	986 (94.2)	1	–
Yes	61 (5.8)	0.70 (0.40–1.23)	.211
PMTCT of HIV			
No	1424 (99.4)	1	–
Yes	9 (0.6)	2.18 (0.58–8.17)	.247
Sickling status			
Negative	1301 (90.8)	1	–
Positive	132 (9.2)	1.29 (0.72–2.33)	.391
Malaria infection			
No	1267 (88.4)	1	–
yes	166 (11.6)	1.19 (0.84–1.70)	.326
Hypertension in pregnancy			
No	1321 (92.2)	1	
Yes	112 (7.8)	1.63 (1.09–2.45)	**.017**
Anemia in pregnancy			
No	483 (33.7)	1	
Yes	950 (66.3)	0.90 (0.70–1.15)	.385
Body Mass Index (BMI) Groups			.164
Underweight	24 (1.7)	1	
Normal weight	670 (46.8)	0.77 (0.33–1.84)	.562
Overweight	443 (30.9)	0.61 (0.25–1.46)	.263
Obese	295 (20.6)	0.85 (0.35–2.06)	.719

Definition: For the purpose of this study “**Grand Multigravida**” was defined as was been pregnant 5 times or more; “**Grand Multiparity**” was defined as 7 times or more deliveries. PMTCT = prevention of mother-to-child transmission, HIV = human immunodeficiency.

### 3.4 Risk factors of prenatal depression among pregnant women

Variables showing *P* ≤ .7 in the bivariate analysis were used for a multivariate logistic regression analysis, which presented estimates of association between independent variables and prenatal depression prevalence. The results showed that advance maternal age ≥35years (AOR 1.49, 95% CI 1.05–2.11, *P* < .026); low educational attainment (AOR 2.15, 95% CI 1.23–2.34, *P* < .007); nulliparous women (AOR 4.93, 95% CI 1.60–15.16, *P* < .005); primiparous women (AOR 5.42, 95% CI 1.76–16.71, *P* < .003); Multiparous women (AOR 4.79, 95% CI 1.61–14.22, *P* < .005) and hypertension in pregnancy(AOR 1.71, 95% CI 1.12–2.60, *P* < .013), were all significantly higher among women with parental depression as shown in Table 3.

**Table 3:** Multivariate logistic regression analysis of factors associated with prenatal depression (N = 1433).

Variables	AOR	95% CI		*P* value
Age in years				
<25	1	–	–	
25–34	0.849	0.645	1.118	.245
≥35	1.490	1.050	2.114	**.026**
Level of education				
Tertiary	1	–	–	
Senior High School	0.762	0.396	1.466	.416
Junior High School	1.233	.707	2.150	.460
Primary & below	2.151	1.230	3.762	**.007**
Employment status				
Salary worker	1	–	–	
Trader	0.785	0.427	1.443	.435
Apprenticeship	0.535	0.286	1.002	.051
Farmer	0.685	0.340	1.383	.291
Hairdresser	0.997	0.463	2.146	.994
Unemployed	0.798	0.425	1.500	.484
Gestational age				
<28weeks	1	–	–	
≥28weeks	0.885	0.678	1.156	.370
Gravida				
Primigravida	1	–	-	
Multigravida	0.802	0.568	1.132	.210
Grand multigravida	0.742	0.495	1.113	.150
Parity				
Nulliparity	4.928	1.603	15.157	**.005**
Primiparity	5.420	1.758	16.712	**.003**
Multiparity	4.785	1.609	14.228	**.005**
Grand multiparity	1	–		
Blood type				
Type A	1	–	–	
Type B	1.128	0.707	1.800	.614
Type AB	0.972	.524	1.804	.929
Type O	0.985	0.673	1.443	.939
Syphilis status				
No	1	–	–	
Yes	1.408	0.723	2.742	.314
Hepatitis B surface antigen (HBsAg) status				
No	1			
Yes	.545	0.288	1.032	.062
PMTCT of HIV				
No	1	–	–	
Yes	2.634	0.543	12.768	.229
Sickling cell status				
No				
Yes	0.872	0.507	1.500	.621
Malaria infection				
No	1	–	–	
Yes	1.447	0.888	2.358	.138
Hypertension				
No	1	–	–	
Yes	1.709	1.121	2.604	**.013**
Anemia				
No	1	–	–	
Yes	0.898	0.695	1.160	.410
Body Mass Index (BMI) group				
Underweight	1	–	–	
Normal weight	0.848	0.345	2.081	.719
Overweight	0.672	0.270	1.671	.393
Obese	0.936	0.374	2.344	.887

Definition: For the purpose of this study “**Grand Multigravida**” was defined as was been pregnant 5 times or more; “**Grand Multiparity**” was defined as 7 times or more deliveries. PMTCT = prevention of mother-to-child transmission, HIV = human immunodeficiency.

## 4 Discussion

### 4.1 Main findings

The estimated prevalence of prenatal depression in the current study was 26.9% (95% CI; 24.6%–29.2%). After eliminating variables with *P* value ≥.05, prenatal depression was found to be associated with advance maternal age ≥35years, low educational attainment (primary school or no formal education), parity (nulliparity, primiparity, or multiparity) and hypertension during pregnancy.

### 4.2 Comparison with other studies

The prevalence of prenatal depression(26.9%) in the current study was high, similar to the 28.7% prevalence of antenatal depression reported in Sodo district, south central Ethiopia,^[24]^ 24.5% reported in Abeokuta North Local Government Area, Nigeria^[25]^ and a pooled prevalence rate reported in Ethiopia (24.2%, 95% CI: 19.8–28.6).^[26]^ However, the prevalence found in our study is shown to be higher than the 21.0% reported for pregnant women in Western Cape, South Africa,^[27]^ 14.8% reported in State of South Minas Gerais, Brazil^[28]^ and the 19.0% reported in Blantyre district, Malawi,^[29]^ but lower than 34.4%% prevalence reported among women in Kintampo Health Research Centre of Ghana by Weobong et al,^[19]^ 35.0% reported in South Africa^[30]^ and the 38.4% reported in Kenya.^[31]^ In addition, various figures have also been given for the prevalence of antenatal depression in the globe. A systematic review and meta-analysis found a combined prevalence rate of 24.3% in South Asia (95%CI 19.03–30.47). The prevalence rates for India (17.74%, 95% CI 11.19–26.96) and Sri Lanka (12.95%, 95% CI 8.29–19.68) were lower than our prevalence levels, while the prevalence rates for Pakistan (32%, 95% CI 23.11–42.87) and Nepal (50%, 95% CI 35.64–64.36) were higher than our study's prevalence rate.^[32]^ The differences between studies can be explained, at least in part, by differences in the population, location and design of the study and the size of the sample.

Our result suggests that older maternal age is associated with prenatal depression symptoms. This current finding is consistent with previous studies suggesting an increased risk of depression in advance maternal age women.^[33,34]^ A similar result was found in a study conducted by Weobong et al,^[35]^ which recorded an increase in maternal age and a significant impact on the development of prenatal depression. Reasons for this correlation can be due to the fact that advanced maternal age women experience higher rates of pregnancy complications, multiple births, obstetrical intervention and extreme maternal morbidity than younger mothers.^[36]^ However, no significant association between older age and prenatal depression was found in other studies.^[37–39]^

The results of this study are consistent with a growing body of evidence suggesting an increased risk of depression in women with low educational attainment.^[40,41]^ Similar results have been identified in other studies conducted by Bunevicius et al^[42]^ and Jeong et al,^[43]^ which reported low education and its major effect on prenatal depression. Backed by others, prenatal depression tends to be more prevalent in women with low educational attainment.^[44,45]^ This increased risk can be due to a number of factors, such as the belief that, unlike low education, higher education is correlated with more productive jobs and higher wages. Such jobs provide economic opportunities and work conditions that help to alleviate financial stress, encourage healthier lifestyles, and thus improve mental health. For example, a cross-sectional study in Osasco, São Paulo, Brazil, found that higher incomes and the level of education of expectant couples were independently associated with lower prevalence of depression during pregnancy.^[46]^ Again, low education may have a detrimental impact on the individual development of a sense of superiority and self-efficacy, which in turn worsens the capacity of people to cope with the challenges and stresses of life.^[47]^ Increasing maternal knowledge and awareness of prenatal depression and its coping strategies could be one of many likely interventions to protect pregnant women from depression in the study area.

We found that multiparous, primiparous, and nulliparous women were independently associated with prenatal depression. The finding of higher risk of depression for multiparous women is supported by some studies.^[34,48,49]^ De Jesus Silva et al^[28]^ reported that women with a higher number of births and children had depression. This was also confirmed in the South African study^[50]^ in which pregnant women with depression also had more children. In addition, nulliparity or primiparity was significantly associated with prenatal depression, which is consistent with the 3 previous studies, which showed that nulliparous or primiparous women are at higher risk than multiparous women.^[33,51,52]^ Others did not, however, found any significant association between parity and depression.^[38,53,54]^

In our study, we found that women with hypertension during pregnancy were 1.68 times more likely to experience prenatal depression than those without such a diagnosis. Our study is parallel to a prospective population-based study conducted by Kurki et al to investigate nulliparous Finnish women receiving prenatal care in the metropolitan area of Helsinki who reported 2.5times an increased risk of depression for preeclampsia.^[55]^ Compared to non-depressed women, those with moderate depression had a 2.3-fold increase in preeclampsia risk, while moderate-severe depression was associated with a 3.2-fold increase in preeclampsia risk as reported by Qiu et al in a case-control study of Peruvian women which is similar to our finding.^[56]^ For example, in a study involving 452 psychiatric outpatients diagnosed with depression, Rabkin et al^[57]^ found that hypertension was 3 times more prevalent than those without hypertension. A study conducted by Jokisalo and colleagues^[58]^ found that a feeling of hopelessness, frustration with treatment, and perceived tension with blood pressure measurement are associated with poor blood pressure control.

### 4.3 Study strengths and limitations

The validated scale used to measure symptoms of depression had good psychometric characteristics. This study provided us with up-to-date information on prevalence and risk factors for prenatal depression and could help in the design of a cohort study in the study area. The results of this study are also useful for public health planning, monitoring, and evaluation. The sample size of this study is among the largest in Ghana and sub-Saharan Africa as a whole. However, due to the nature of the design of the study, in general, and the fact that this is a one-time measurement of exposure and outcome, it is difficult to establish a causal relationship. Similarly, sampling bias may have happened as a result of respondents who decline to participate in a study. For example, if pregnant women with depression were less likely to engage in the study than those without depression, the frequency of depression in the area could have been underestimated. Furthermore, while systematic sampling, as utilized in current study, decreases the likelihood of sample bias, it does not totally remove it. A biased sample may result if the sampling frame, or the actual list of pregnant women from whom the sample was drawn, does not match the population. Again, one of the selected hospitals was involved and this could limit the generalizability of the findings of the study. Therefore, this study may not be sufficient to understand the trends of the disease in this situation. Although depressive symptoms were evaluated using a valid self-administered scale, psychiatrists did not perform any further clinical assessments to evaluate depression among the participants in this study.

### 4.4 Implications

Our study findings have contributed to an understanding of antenatal depression and the importance of alertness in mental health and well-being during pregnancy. The findings recorded in this study also indicate that attempts should be made to routinely identify pregnant women who are depressed and to use the information to improve the mental health status of the affected population. Identification of prenatal depression and its risk factors help health care professionals to monitor high-risk women and to administer preventive interventions and careful treatment. Future studies and clinical efforts need to shift towards successful cost-effective antenatal depression intervention.

## 5 Conclusion

Depression during pregnancy is high in the study area and significantly associated with advanced maternal age, low educational attainment, parity less than 7 deliveries and hypertension during pregnancy. Screening for depression needs to be incorporated with antenatal care services particularly for women at risk of prenatal depression for early detection and successful intervention.

## Acknowledgments

We would like to thank the women who participated in the study. We would like to thank the research data team, the antenatal, and administrative staff of the Adventist Hospital in Bekwai Municipality for their support for the study. We appreciate the recommendations made by the representatives of the International Advisory Board of Central South University, the Department of Epidemiology and Health Statistics of the Xiangya School of Public Health and thank our collaborators.

## Author contributions

**Conceptualization:** Kwabena Acheampong.

**Data curation:** Kwabena Acheampong.

**Formal analysis:** Kwabena Acheampong, Xiongfeng Pan, Aizhong Liu.

**Funding acquisition:** Aizhong Liu.

**Investigation:** Kwabena Acheampong.

**Methodology:** Kwabena Acheampong.

**Project administration:** Kwabena Acheampong.

**Resources:** Xiongfeng Pan, Atipatsa Chiwanda Kaminga.

**Supervision:** Atipatsa Chiwanda Kaminga, Aizhong Liu.

**Validation:** Xiongfeng Pan, Atipatsa Chiwanda Kaminga, Aizhong Liu.

**Visualization:** Xiongfeng Pan, Aizhong Liu.

**Writing – original draft:** Kwabena Acheampong.

**Writing – review & editing:** Kwabena Acheampong, Xiongfeng Pan, Aizhong Liu.
